# Sources of Pathogenic Nucleic Acids in Systemic Lupus Erythematosus

**DOI:** 10.3389/fimmu.2019.01028

**Published:** 2019-05-08

**Authors:** Tomas Mustelin, Christian Lood, Natalia V. Giltiay

**Affiliations:** Division of Rheumatology, Department of Medicine, University of Washington, Seattle, WA, United States

**Keywords:** lupus, interferon, nucleic acid sensors, mitochondria, reverse transcriptase

## Abstract

A hallmark of systemic lupus erythematosus (SLE), and several related autoimmune diseases, is the presence of autoantibodies against nucleic acids and nucleic acid-binding proteins, as well as elevated type I interferons (IFNs), which appear to be instrumental in disease pathogenesis. Here we discuss the sources and proposed mechanisms by which a range of cellular RNA and DNA species can become pathogenic and trigger the nucleic acid sensors that drive type I interferon production. Potentially SLE-promoting DNA may originate from pieces of chromatin, from mitochondria, or from reverse-transcribed cellular RNA, while pathogenic RNA may arise from mis-localized, mis-processed, ancient retroviral, or transposable element-derived transcripts. These nucleic acids may leak out from dying cells to be internalized and reacted to by immune cells or they may be generated and remain to be sensed intracellularly in immune or non-immune cells. The presence of aberrant DNA or RNA is normally counteracted by effective counter-mechanisms, the loss of which result in a serious type I IFN-driven disease called Aicardi-Goutières Syndrome. However, in SLE it remains unclear which mechanisms are most critical in precipitating disease: aberrant RNA or DNA, overly sensitive sensor mechanisms, or faulty counter-acting defenses. We propose that the clinical heterogeneity of SLE may be reflected, in part, by heterogeneity in which pathogenic nucleic acid molecules are present and which sensors and pathways they trigger in individual patients. Elucidation of these events may result in the recognition of distinct “endotypes” of SLE, each with its distinct therapeutic choices.

## Introduction

Systemic lupus erythematosus (SLE) is a serious autoimmune disease characterized by autoantibodies against nucleic acids and nucleic acid-binding proteins combined with immune complex deposition and inflammatory manifestations in multiple organ systems. The unpredictable course of the disease with its sudden exacerbations, often with new organ manifestations or symptoms, make it particularly difficult to manage (1, 2), not the least because the currently available drugs have limited efficacy and/or serious side-effects. Efforts to develop more selective and more efficacious therapies that address the core pathobiology of SLE, ideally with limited general immune suppression, continue to be hampered by our limited understanding of the underlying molecular drivers and mechanisms (3). To vividly illustrate this, the two newest therapeutics for SLE are hydroxychloroquine (4, 5) and belimumab (6, 7), approved by the FDA in 1966 (sic!) and 2011, respectively. Moreover, the latter had barely significant efficacy, only 9.8% SLE Responder Index improvement over placebo at 52 weeks at the highest 10 mg/kg dose (6). Furthermore, while the presence of autoantibodies in SLE has been recognized for decades and their role in driving disease is considered well established, B cell depletion by anti-CD20 antibodies have failed to generate statistically significant efficacy in clinical trials in SLE (8). There is, however, a trend toward a benefit for patients in agreement with the ability of belimumab to reduce B cell numbers. It should also be noted that belimumab may affect plasma cells more than the depletion of CD20-positive B cells. T cell-directed therapies, such as calcineurin inhibitors (9) or CD28 blockade with CTLA4-Ig (10), have also yielded limited disease impact. These outcomes suggest that many of the well-documented immune abnormalities in SLE may be consequences, rather than drivers, of this disease.

## Autoantibodies Against Nucleic Acids and Nucleic Acid-Binding Proteins

In SLE, the majority of patients develop autoimmunity toward nuclear antigens, conveniently measured as anti-nuclear autoantibodies (ANA). Though not selective for SLE, detecting ANA is a common test used to screen patients, and may, together with clinical presentation and other immunological features, suffice for SLE diagnosis. ANA contains a broad range of autoantibodies targeting among others chromatin, histones, double-stranded (ds) DNA, as well as the RNA-binding proteins Ro, La, Sm, and RNP. Anti-dsDNA antibodies are of particular interest in SLE, given their high diagnostic potential, with about 70–80% of the patients being positive for these antibodies, and titers commonly correlating with disease activity. Indeed, anti-dsDNA antibodies have been included in the classification criteria (11), as well as a serological component of the disease activity index SLEDAI (12). Further, anti-dsDNA antibodies are often associated with severe disease manifestations, including nephritis. Other than the diagnostic value, including associations with distinct disease features, these autoantibodies may be pathogenic through immune complex-mediated inflammation, complement activation and tissue destruction, and antibody-directed cellular cytotoxicity. In this review, we will limit our discussion of autoantibodies to their ability to transport nucleic acids, shielding them from external nucleases, and efficiently mediating their uptake into immune cells through Fc receptors, complement receptors, scavenger receptors, and others.

## The “IFN Signature” in SLE Patients

In 2003, Tim Behrens' group (13), the team of Virginia Pascual and Jacques Banchereau (14), and Mary Crow (15) published their discovery that SLE patient blood contain active type I interferon (IFN) and a high expression level of IFN-stimulated genes (ISGs), now referred to as the “IFN signature.” Although indications that IFNα may be important in the lupus pathogenesis had been published earlier (16–18), this still was a surprising finding because the principal function of type I IFN is in host defense against viral infection, while SLE is not an infectious disease. Nevertheless, the IFN signature is now a well-established observation in 70–90% of SLE patient populations world-wide (19–22). Individual IFNs are technically difficult to measure (23) due to their very low concentrations and presumed rapid consumption, but it seems that many of the 17 different type I IFNs, which includes 13 IFNα isoforms, IFNβ, and the three less explored members, IFNε, IFNκ, and IFNω are elevated in SLE patients, as well as in patients with Sjögren's syndrome (24, 25), systemic sclerosis (26, 27), polymyositis, dermatomyositis (28, 29), rheumatoid arthritis (30, 31), and other related diseases. Importantly, there seems to be differences between patients in which specific members of the type I IFN family are elevated (see sections Patient heterogeneity with regard to nucleic acids and their sensors? and Can SLE be divided into clinically meaningful subpopulations based on “endotype”?). In addition, patients may have increased type II IFN (IFNγ) (25) and/or type III IFNs (IFNλ1, IFNλ2, and IFNλ3, also known as IL-29, IL-28A, and IL-28B) (32). While the type I and III IFNs are functionally overlapping (all genes induced by type III IFNs are also induced by type I IFNs), IFNγ is instrumental in a distinct aspect of the immune system, namely the activation of CD4 Th1 and CD8 T cells, natural killer (NK) cells, and other elements of a general immune response. Nevertheless, over 900 of the 1,300 ISGs induced by IFNγ are also induced by type I IFNs, which induces a total of over 1,500 ISGs, suggesting significant overlap in downstream consequences.

Type I IFNs have a spectrum of effects on the immune system and beyond, particularly upregulating numerous mechanisms of on anti-viral defense. They stimulate emergency myelopoiesis (33), monocyte differentiation into myeloid dendritic cells (34, 35), antigen presentation, cytotoxic T cell differentiation (36), and B cell differentiation into plasma cells (37). The 1,500 ISGs encode many immune-modulating as well as direct antiviral proteins (38), including many components of the pathways that lead to type I IFN production in what constitutes a rapid positive feedback loop to augment the response.

While an extensive literature illuminates the close association of type I IFNs with SLE pathogenesis and disease activity (21, 39), perhaps the most conclusive evidence for a causal role in the disease was the statistically significant efficacy in phase 2 clinical trials (40) of an antibody that blocks the type I IFN receptor used by all type I IFNs. In contrast, an antibody that blocks IFNα alone (41) was efficacious only in a small subset of patients. It should also be noted that blocking the type I receptor did not bring clinical improvement to all SLE patients even if the IFN signature declined by over 90% in the treated patient population. Nevertheless, elevated type I IFNs are the closest we have to a smoking gun in SLE and a set of related autoimmune diseases. This, in turn, begs the question: why are type I IFNs elevated in SLE patients?

## Nucleic Acid Sensors Coupled to Interferon Production

**Figure 1:**
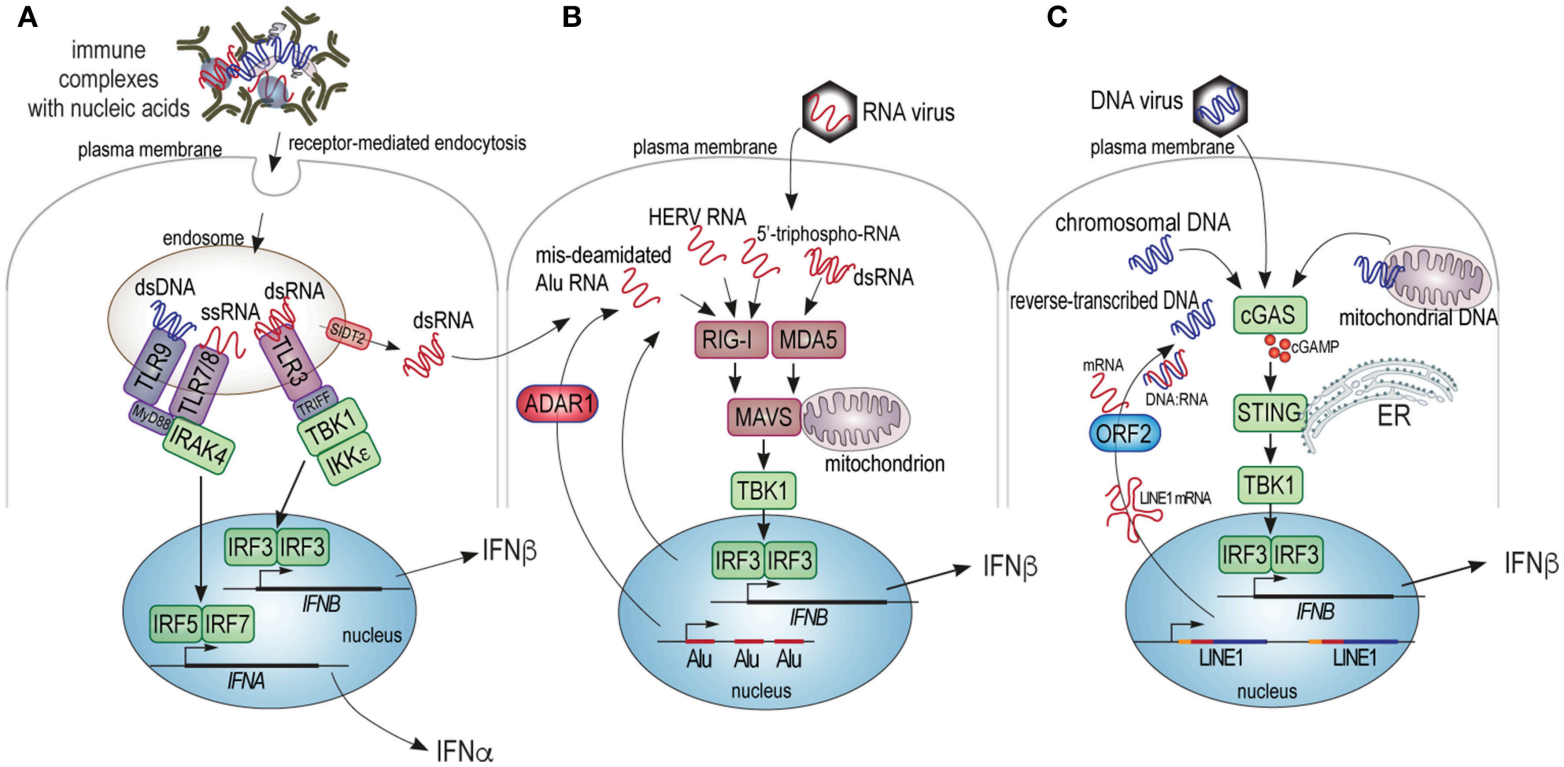
Schematic illustration of the cellular sensors of pathogenic DNA and RNA and their signaling pathways leading to type I IFN production. **(A)** Extracellular nucleic acids present in immune complexes, free mitochondria, or other structures can be internalized into cells by receptor mediated endocytosis and trafficked to endosomes that contain TLR3, 7, 8, or 9, which recognize dsRNA, ssRNA, and dsDNA, respectively. TLR3 signals through the TRIFF adapter and the protein kinases TBK1 and IKKε, which phosphorylate and activate the IRF3 transcription factor, which (with co-factors) transactivates the gene for IFNβ. TLR7, 8, and 9 signal through the MyD88 adapter and the IRAK4 protein kinase to primarily phosphorylate and activate transcription factors IRF5 and IRF7, which participate in the transactivation of some or all of the 13 genes for isoforms of IFNα. Finally, the SIDT2 transporter in the endosome membrane can mediate the exit of dsRNA into the cytosol of the cell to be sensed by MDA5. **(B)** Cytosolic RNA from exogenous viruses, or endogenous transcripts improperly deaminated by ADAR1, or containing recognizable retroviral motifs (HERV RNA), or potentially other aberrant RNA species can trigger RIG-I or MDA5, which principally bind ssRNA and dsRNA, respectively. Upon ligand binding RIG-I or MDA5 trigger the oligomerization of the MAVS protein, which assembles a protein complex on the mitochondrial membrane, resulting in activation of the TBK1 protein kinase, which activates IRF3. **(C)** Cytosolic DNA from exogenous viruses, pieces of chromatin, mitochondrial DNA, or reverse-transcribed RNA, triggers dimerization and activation of cGAS leading to the synthesis of cGAMP, which activates the STING adapter on the surface of the endoplasmic reticulum (ER). STING, in turn, activates the TBK kinase, which activates IRF3.

Given that the best recognized role of type I IFN is in defense against viral infection (38), it seems that one could find important clues about the upstream mechanisms of SLE from recent advances in viral immunity. The primary threat that a virus brings is its RNA or DNA genome, which will hijack the cellular biosynthetic machinery for its own replication and virion production, with detrimental consequences for the host cell. Even more alarming, retroviruses will reverse transcribe their RNA genome and insert the resulting DNA into the host genome as a permanent provirus. To combat these ancient foes, evolution has produced several cellular mechanisms for the detection of non-self RNA and DNA (Figure 1). Four principal pathways operate in the cytosol and on the surface of intracellular organelles: the DNA-sensor “cyclic GMP, AMP synthase” (cGAS) (42), the RNA sensors “retinoic acid-inducible gene I” (RIG-I) (43), “melanoma differentiation-associated gene 5” (MDA5) (43–45), and “RNA-activated protein kinase” (PKR) (46, 47), while a fifth pathway responds to extracellular DNA or RNA brought into the cell by receptor-mediated endocytosis and is initiated by Toll-like receptors (TLRs) 3, 7, 8, and 9 in the endosomal compartment. A mechanism to blend the extracellular and intracellular sensing pathways was recently reported (48): the transporter protein SIDT2 in the endosomal membrane functions to let dsRNA escape the endosome into the cytosol, where it can trigger MDA5. There are additional, more recently discovered nucleic acid sensors, such as DDX1, 21, 36, and 41, IFI16, and Aim2 (49). All of these pathways lead to type I IFN production through activation of IRF3 and related transcription factors. They also activate other signaling pathways that lead to the production of additional cytokines. The resulting type I IFNs are secreted, bind to the type I IFN receptor, and signal through the JAK/STAT pathways to upregulate ISG-encoded proteins with direct anti-viral activity, including nucleases, helicases, chaperones, and many of the sensors and their adapters and signaling proteins (38). Type I IFN can act in both autocrine and paracrine fashion and the response to them may differ between different responding cell types.

Whereas, nucleic acids are the main triggers of type I IFN production, the cell type producing them and the exact nature of the triggering nucleic acid will determine which type I IFNs are produced. For example, plasmacytoid dendritic cells (pDC) have a particularly high capacity to produce several isoforms of IFNα in response to viruses or immune complexes that contain nucleic acids (50–52), including those containing IgE (52), by a TLR7 or 9-dependent mechanism. Non-immune cells, on the other hand, tend to produce predominantly IFNβ in response to cytosolic RNA or DNA through the sensors MDA5 (dsRNA), RIG-I (RNA), and cGAS (dsDNA), with other sensors participating, particularly in neutrophils that do not express cGAS (53).

### TLRs in SLE

TLRs are central to the immune system's ability to recognize molecular structures associated with cellular damage or pathogens (54), including nucleic acids by TLRs 3, 7, 8, and 9 (Figure 1A). Since their discovery over 20 years ago, much of the early literature assumed that their role in SLE was certain (55–58), particularly since their ligation triggers type I IFN production and circulating immune complexes that contain nucleic acids are present in abundance in most SLE patients (59), as well as in the patients with related diseases like Sjögren's syndrome, polymyositis, dermatomyositis, mixed connective tissue disease, and others. It still seems very likely that these immune complexes drive production of IFNα by plasmacytoid dendritic cells (pDC) primarily through activation of TLR7 and maybe 9 (60). However, so far all tested antagonists of TLR 7 and/or 9 (61) have failed to provide any efficacy in placebo-controlled clinical trials in SLE patients. If type I IFNs indeed are important, but TLR7 and 9 inhibition does not produce a therapeutic benefit, then reality must be more complex. Indeed, the more recent discovery of other sensors for nucleic acid, such as cGAS, RIG-I, and MDA5 introduced other options for nucleic acid sensing leading to type I IFN. Nevertheless, it still seems likely that TLR7/9 drive IFN production in response to circulating immune complexes that contain nucleic acid and thereby contribute to the IFN signature seen in SLE patients. Unfortunately, clinical trials with TLR antagonists did not report what effects these drugs had on the IFN signature.

Recent advances in TLR research has revealed intriguing new details about the mechanisms of their ligand interactions, including their ability to bind self-nucleic acids (62–66). While TLR9 was originally proposed to only sense bacterial DNA with CpG sites, it is now clear that it can also recognize chromosomal and mitochondrial DNA (digested into small fragment by DNase II). Similarly, TLR3 responds to self-derived non-coding RNA, such as U1 RNA that might be released upon cellular stress, including exposure to UV radiation, while TLR7 and 8 can also recognize RNA and DNA degradation products (66). Another recent study found that phagocytosis of anti-dsDNA IgE antibodies (found to be increased in some SLE patients) via the high-affinity FcεRI receptor for IgE, mediates TLR9-mediated sensing of self-DNA in the phagosomes and potentiates IFN production by plasmacytoid dendritic cells (52).

Another potentially important aspect of the TLR pathways is that the *TLR7* and *TLR8* genes are located on the X-chromosome: there are indications that *TLR7* may escape the normal silencing of one of the two X chromosomes in females (67), resulting in higher levels of TLR7 expression and, hence, stronger responses to TLR7 simulation in immune cells in women, perhaps contributing to the 9:1 gender bias in SLE. In further support of a role of TLR7 quantity in the disease, copy number variations (68, 69) and single-gene polymorphisms (70) in *TLR7* are associated with SLE susceptibility.

### Activation of cGAS and RNA Sensors in SLE

A recent paper provided the first direct evidence that the cGAS pathway is activated in at least a subset of SLE patients: the second messenger cyclic-guanine, adenosine-2,3-phosphate (cGAMP), which is synthesized exclusively by cGAS upon DNA binding, was detected by mass spectrometry in 7 of 30 SLE patients (71). While it may seem that this represents a small portion of SLE patients, it is important to recognize that the data represent a single snap-shot in time and that cGAMP is a short-lived second messenger present in minute quantities. Thus, it may well be that cGAMP is elevated in more SLE patients.

Direct evidence for activation of RNA sensors in SLE patients was also reported recently (72). Twenty two of sixty-seven examined SLE patients had evidence of polymerization of the mitochondrial antiviral signaling protein MAVS, which is downstream of both RIG-I and MDA5 (Figure 1B) and acts by generating a protein complex that activates the kinases required for IRF3 activation and type I IFN production. This aggregation of MAVS indicates that either RNA sensor was triggered in 32% of the patients.

## Aicardi-Goutières Syndrome–a Monogenic Disease of Nucleic Acid Processing

Additional insights into the molecular mechanisms that can drive type I interferons and cause interferon-dependent human disease come from a monogenic inherited inflammatory syndrome called Aicardi-Goutières syndrome (AGS) (73–77), which, together with a few related diseases, is included in the concept of the “type I interferonopathies” (78). AGS usually presents neonatally as a suspected serious viral infection with fever, chills, and a failure to thrive, accompanied by high levels of type I IFNs. However, a virus is not detected and the symptoms continue unabated. Over time, AGS patients develop neurological deficits and brain calcifications, likely due to the neurotoxicity of IFNs, as well as systemic autoimmunity with autoantibodies against nucleic acids and nucleic acid-binding proteins very similar to those in SLE patients. In fact, many AGS patients meet the diagnostic criteria for SLE (73–77).

AGS is a caused by mutations in any one of eight genes: *TREX1, RNASEH2A, RNASEH2B, RNASEH2C, SAMHD1, ADAR1, IFIH1, TMEM173* (73–77). The first 5 of these genes are primarily involved in the defense against retroviruses and their endogenous remnants in our genome (79). In fact, many of these genes were first discovered as “restriction factors” by researchers studying how HIV replicates in certain cells, but not in others. The revelation that our genome contains many evolutionarily conserved genes that confer resistance to HIV suggested that HIV is not the first exogenous retrovirus to infect us, but, in fact, is just the latest in a very long series of retroviral infections resulting in germline integrations of numerus families of retroviruses that today constitute as much as 8% of our genome (or as much as ~40% if other retroelements of ancient retroviral origin are also counted).

The three other genes that can induce AGS are homozygous loss-of-function mutations in the gene for “adenosine deaminase acting on RNA 1” (ADAR1), *IFIH1* which encodes MDA5, and the gain-of-function variant of *TMEM173* which encodes constitutively active STING (the direct effector protein for cGAS, Figure 1C) and results in the constitutive activation of this pathway in the absence of aberrant DNA (80). The two latter genes demonstrate that chronic activation of RNA sensing (MDA5) or DNA (cGAS/STING) leads to an SLE-like condition in humans.

## Sources of Type I IFN-triggering DNA and RNA

Since type I IFNs are elevated in most SLE patients and appear to play an important role in SLE pathogenesis and since perturbations in nucleic sensing pathways lead to a disease (i.e., AGS) characterized by chronically elevated type I IFN and autoimmunity with many of the same autoantibodies as in SLE, it seems logical to ask if sensor-triggering nucleic acids might be present in SLE patients. Alternatively, the function or regulation of one or several sensors might be faulty. Although evidence exists for the association of genetic variants of DNA and RNA sensors with SLE, and mutations in them can cause type I interferonopathies (80), such mutations are present in a very small subset of SLE patients. Hence, it would be important to elucidate which nucleic acids are aberrantly present in SLE patient. What is their nature and origin?

Although viruses have long been suspected to play some role in triggering several different autoimmune diseases, there is little evidence for a persistent presence of viral RNA or DNA in SLE patients. If aberrant nucleic acids are present in SLE patients to trigger the DNA and/or RNA sensors discussed above, they likely are derived from endogenous sources, such as chromosomal DNA, mitochondrial DNA, DNA made by reverse-transcription from RNA templates, RNA transcripts from normally silent loci of ancient viral origin (that still somehow resemble viral RNA), mis-edited RNA, or otherwise altered or improperly processed RNA molecules (Figure 1). We will discuss these potential sources one by one.

### Chromosomal DNA

While chromosomal DNA normally is well protected by myriad binding proteins and a highly ordered packing into nucleosomes and higher order structures, DNA damage or faulty DNA replication can, in principle, dislodge smaller pieces of DNA, for example as nuclear blebs or micronuclei found in cancers (81). The existence of several effective DNA repair mechanisms indicate that DNA damage does occur in cells for a variety of reasons, including during normal aging. It is conceivable that DNA damage could produce pieces of DNA that trigger cGAS and subsequent type I IFN production (82, 83). Loss of DNA degradation by DNase1L3 causes an autosomal recessive form of SLE with early life onset and high prevalence of nephritis (84), and loss of the Trex1 DNase (85, 86) also leads to constitutive type I production and SLE or AGS, indicating that rapid elimination of aberrant DNA is important for the maintenance of health.

Cell death, whether by physiological programmed cell death mechanisms, such as apoptosis, or, more likely, by more pathological, inflammatory, or toxic mechanisms like necrosis, pyroptosis, or necroptosis, can result in the release of chromosomal DNA (and RNA) into the extracellular milieu (87, 88). Many protective processes have evolved to minimize this exposure to chromatin and the highly toxic histones (89). Apoptotic cells are recognized by specific receptors for phosphatidylserine, annexin V, and other molecules that serve to mark apoptotic cells to facilitate their rapid removal by tissue macrophages (90) and the reticuloendothelial system. When these mechanisms are faulty or overwhelmed by massive numbers of dying cells, anti-nuclear and nucleic acid-directed autoantibodies and autoimmune disease may develop (87). For example, severe viral infections that are accompanied by immune-mediated killing of large numbers of infected cells typically result in measurable titers of anti-nucleic acid autoantibodies in otherwise healthy individuals. However, these titers tend to be relatively modest and they decline after the infections is cleared. The complement system (91), particularly C1q, also participates in the non-inflammatory removal of dying cells, perhaps explaining why complement deficiencies predispose to SLE (92). C1q also influences type I IFN production by a more direct mechanism (93, 94).

### Mitochondrial DNA

Another source of nucleic acids are the mitochondria (95, 96), which serve many functions besides oxidative phosphorylation and production of ATP, such as metabolism, inflammation and cell death. Though mainly found intracellularly, we recently discovered that neutrophils can extrude mitochondria together with chromosomal DNA during the formation of neutrophil extracellular traps (NETs) (95, 97). The externalization of mitochondria depended on the generation of reactive oxygen species (ROS) and the extruded mitochondria contained highly oxidized (8-OHdG) mitochondrial DNA, inducing IFNβ generation in a process requiring the intracellular DNA sensor adaptor protein, STING (95). Blocking mitochondrial ROS generation *in vivo* ameliorated lupus-like disease in MRL/*lpr* mice (95). These observations appear to be clinically relevant as *ex vivo* neutrophils from SLE patients displayed ongoing mitochondrial ROS production and spontaneous extrusion of oxidized inflammatory mitochondrial DNA (95). Similar to NET formation, as described above, other forms of cell death, such as TNF-mediated necroptosis, have been shown to involve the release of intact mitochondria into the extracellular environment (98, 99). Though the intracellular source(s) of extruded DNA has yet to be verified in other forms of cell death, e.g., pyroptosis, we find it likely that any form of cell death that includes breakdown of the plasma membrane will result in the release of mitochondria or their components, such as mitochondrial DNA.

Mitochondria and mitochondrial DNA may also be released from live cells upon their activation, as shown in neutrophils, eosinophils, mast cells and platelets (100–103). In neutrophils, this process has been coined “vital” NETosis, as the neutrophil remains alive after the extrusion event. Circulating platelets are thought to be the primary source of cell-free mitochondria given the large abundance of platelets in blood. Work from the laboratory of Eric Boilard has demonstrated that platelets, upon activation, may extrude naked mitochondria able to undergo respiratory burst (103). Unless these mitochondria are rapidly cleared, secreted phospholipase A2 will hydrolyze and weaken the mitochondrial membrane, causing disruption and the release of inflammatory mitochondrial DNA and other damage-associated molecules (103). Platelet-mediated extrusion of mitochondria can occur in concentrated platelet preparations and is associated with adverse reactions upon transfusions (103, 104). Mitochondria can also be released as part of microparticles from many different cells, including platelets, neuronal and glial cells, as well as hepatocytes (103, 105, 106). The role of platelet-mediated release of mitochondrial DNA is of particular interest in rheumatic disease, including SLE, given the marked platelet activation and subsequent development of cardiovascular morbidity and mortality observed in these patients (107–110).

While the role of mitochondrial extrusion and mitochondrial DNA (oxidized or not) in SLE remains to be clarified, it is clear that they can be derived from many different cells and involve either the activation or death of these cells. It also appears that the DNA sensors TLR9 (105, 111–113) and cGAS (95, 114, 115) can be triggered by mitochondrial DNA, presumably depending on its subcellular location or pathway of receptor-mediated internalization (Figure 1). Elevated amounts of free mitochondrial DNA have been observed in several conditions, including chronic inflammatory diseases, trauma, cardiovascular disease and rheumatoid arthritis, perhaps promoting inflammation and even mortality (103, 116–118). Further studies to elucidate the mechanisms by which extruded mitochondria and/or mitochondrial DNA are cleared will be important for our understanding of this biology and for the design of therapeutic regimens to prevent the contribution of mitochondria and/or their DNA to human autoimmunity.

### Reverse-Transcribed RNA

The third source of DNA that may trigger type I IFN synthesis is intracellular DNA made by the reverse transcription of cellular RNA. Our genome encodes three different families of reverse transcriptases (RTs): telomerase (*TERT*), the *pol* genes of many endogenous retroviruses, and the second open-reading frame (ORF2) of the long interspersed nuclear element-1 (LINE1). Of these enzymes, telomerase is highly specialized to synthesize TTAGGG repeats in the 194 telomeres of our diploid chromosomes using the *TERC* RNA template (119, 120), while retroviral RTs only function to convert the RNA genome of an incoming retrovirus to a DNA provirus and to insert it into the genome. Although our genome contains thousands of endogenous retroviral provirus loci, none of them appear to be infectious anymore (with the possible exception of HERV-K113). This leaves only the LINE1 ORF2 enzyme as a candidate RT capable of generating aberrant DNA that could trigger type I IFN production through cGAS activation. It has been demonstrated to have robust RT activity (121–123), which is key for retrotransposition (124) and which is sensitive to some clinically used RT inhibitors (125, 126).

The LINE1 element represents a remnant of an ancient retrovirus that retained, or later acquired, a degree of autonomy through the conservation of a primordial RT, which endows it with the ability to transpose by a “copy and paste” mechanism. The LINE1 RNA transcript is 6 kb long and contains two open-reading frames (79): ORF1, which encodes a 40-kDa RNA-binding protein that co-localizes with LINE1 mRNA in stress granules together with other RNA-binding proteins (127), such as Ro60, La, and U1 Small nuclear ribonucleoprotein of 70 kDa (127), and ORF2, which encodes a 150-kDa RT and endonuclease. Attesting to the effectiveness of the LINE1 element's ability to transpose, there are over half a million copies of it throughout our genome. However, due to defense mechanisms (including those encoded by the AGS genes) and mutational rate, the vast majority of these copies are truncated and mutated and no longer have the ability to transpose. It has been estimated that < 180 LINE1 copies are seemingly intact, but that only 5 or 6 of them are active (“hot”) today (128). The LINE1 ORF2-encoded RT is also involved in generating and transposing Alu element copies (129) and was, over evolutionary time, responsible for generating all of our processed pseudogenes (130). In other words, the LINE1-encoded RT has had a profound impact on our genome and our health.

The study of AGS revealed that transcription of retroelement loci is very low in healthy individuals, but that AGS patients have elevated levels of retroelement mRNAs and proteins, including enzymatically active LINE1 RT (79). In fact, LINE1 RT may be the main producer of pathogenic DNA that triggers type I IFN production (131) in AGS patients with *TREX1, RNASEH2A, RNASEH2B, RNASEH2C*, and *SAMHD1* mutations. TREX1 is the DNase that degrades intracellular DNA made by reverse transcription (86, 132), including DNA in complex with RNA as occurs during reverse transcription, while RNaseH2 preferentially acts on the RNA in such heteroduplexes (77). Finally, SAMHD1 is a phosphohydrolase specific for the deoxynucleotide triphosphates (dATP, dTTP, dGTP, and dCTP) required for reverse transcription (133). In a mouse model of AGS, the *Trex1*^−/−^ mouse (86), the animals develop a systemic inflammation with immune cell infiltrates in many organs and they die early from a severe carditis. These animals can be rescued from death by treatment with the RT inhibitors tenofovir plus nevirapine (134), indicating that reverse transcription is a key step in the pathogenesis of systemic inflammation in this model. However, there is also a published paper refuting these data (135). A human clinical trial with RT inhibitors in AGS is under way.

There is some evidence that LINE1 retroelements are activated also in SLE patients (136–138). This appears to correlate with a global decrease in DNA methylation, which is well documented in SLE (139, 140) and likely relates to the decreased expression of DNA methylases DNMT1 and DNMT3a (141, 142). Demethylating agents like 5-aza-2′deoxycytidine (143) also cause a dramatic upregulation of LINE1 and Alu element transcription in lymphocytes (144). In addition, transfer of 5-aza-2′deoxycytidine-treated T cells into healthy mice results in an SLE-like disease (145). The drugs that can induce “drug-induced lupus,” notably hydralazine and procainamide, are demethylating agents (146). Other known triggers of lupus flares, like UV light, oxidative stress, inflammation and exogenous viruses also induce genomic hypomethylation (147, 148).

We are aware of only two papers that report the detection of LINE1-encoded ORF1 and ORF2 proteins in samples from patients with SLE or related diseases. Mavgrani and co-workers (136) showed by immunoblotting and immunohistochemistry that p40/ORF1 was readily detectable in kidney samples from lupus nephritis patients and in salivary gland biopsies of Sjögren's patients. Staining correlated with IFNβ in somatic cells and IFNα in infiltrating plasmacytoid dendritic cells. As activation of LINE1 elements in autoimmune patients (136–138) appears to involve demethylation of the LINE1 promoter (136, 138, 140), these authors also analyzed the methylation of CpG sites in the LINE1 promoter and found it to be reduced in patients with elevated LINE1 expression. In the second paper, Kalogirou et al. demonstrated that ORF2 is upregulated in the ductal cells of salivary gland biopsies from patients with Sjögren's syndrome (149).

### Cellular RNAs and RNA Editing by ADAR1

Many viruses have an RNA genome and do not (unlike the retroviruses) generate any DNA. A set of cellular RNA sensors have evolved to detect these viruses (150) (Figure 1B), a challenging task given the abundance of cellular RNA species. It remains incompletely understood how these sensors can distinguish between self and foreign RNA molecules, but the length of double-stranded RNAs (150) and capping modifications of the 5′ and 3′ ends of RNA molecules (151), as well as the presence or absence of other types of RNA processing, appear to matter. The delicate balance between the recognition of self- vs. foreign RNA is well illustrated by the *IFIH1*-A947T allele, which encodes a variant of MDA5 that enhances anti-viral immunity, but increases the risk of autoimmunity (152, 153).

Extracellular RNA, for example in immune complexes with the Ro protein (154), can also enter immune cells via receptor-mediated endocytosis followed by trafficking to the endosomal compartment where TLR3 will react to dsRNA and TLR7 and 8 with single-stranded RNA (as well as other ligands, see section TLRs in SLE). From this compartment, dsRNA may also exit into the cytosol through the SIDT2 channel (48) to trigger cytosolic MDA5. This pathway likely exists to aid in the detection of RNA viruses that are captured by antibodies, complement, or scavenger receptors.

The role of ADAR1 is also very interesting as this enzyme is involved in the post-transcriptional editing of mRNAs by converting adenosine to inosine, which is read as a guanosine during translation. Interestingly, in humans (unlike other organisms) the majority of the deaminated adenosines are non-coding and located in RNA molecules derived from Alu elements and other transposable sequences (155). The induction of type I IFN by mutated ADAR1 is dependent on MDA5, but not on RIG-I, suggesting that RNA editing is important and that its absence triggers the MDA5 pathway as if viral dsRNA was present.

### Endogenous Retroviral RNA

While exogenous viruses introduce RNA (or DNA) molecules that can be recognized as foreign by cellular sensors, it remains doubtful that RNA transcripts from endogenous proviruses (which constitute as much as 8% of our genome) would be seen as foreign as they are transcribed and processed by the normal cellular machinery. Nevertheless, since these sequences are of viral origin, it is possible that some of them still contain sequence motifs that allow cellular RNA sensors to recognize them as non-self. If so, one would expect the relevant loci to be among the most recently incorporated ones, which may not yet have accumulated domesticating mutations. Furthermore, since most people do not develop autoimmunity, one would also assume that they normally are effectively silenced in healthy individuals, but perhaps aberrantly expressed in patients with SLE or related diseases.

### Transposable Element RNA

A much more interesting category of RNAs in autoimmunity research are those encoded by Alu elements and other short transposable elements, not perhaps because of their origin, but because they have been experimentally implicated in several settings. An important paper in this respect reported that a large portion of all RNA present in circulating anti-Ro autoantibody immunocomplexes was Alu element RNA (154). In fact, other SLE autoantigens, such as La (156), also bind Alu RNA. Furthermore, Ro^−/−^ mice (157) develop autoimmunity resembling SLE, suggesting that the normal function of Ro is important for preventing the Alu element RNA, and perhaps other cellular RNA molecules (158, 159), from triggering RNA sensors. The discovery that the RNA-editing enzyme ADAR1 primarily edits Alu transcripts in humans (155) and that the LINE-1 encoded RT has catalyzed the reverse transcription and genomic insertion of over a million copies of the Alu element in our genome, as well as the co-localization of LINE1 proteins with Ro and La, hints at a central, but still enigmatic, role of this RNA biology in SLE pathogenesis. It also remains unknown how Ro-Alu RNA complexes end up in the extracellular compartment, but one can suspect that cell death by several programmed mechanisms must be involved.

## Patient Heterogeneity With Regard to Nucleic Acids and Their Sensors?

While it seems likely that type I IFNs play an important role in the pathogenesis of SLE and related diseases, it also becoming clear that their inhibition is not a cure for all patients. For example, 10–30% of SLE patients do not have an IFN signature, suggesting that their disease does not involve elevated type I IFNs and may therefore be molecularly altogether different. Furthermore, within the subpopulation of SLE patients with an IFN signature, therapeutic antibodies that neutralize IFNα (41, 160, 160, 161) or all type I IFNs (by blocking their receptor) (40) have been clinically efficacious in some patients, but not in others. The reasons for this heterogeneity are not understood, but may be related to the complexity of the interferon system, the coverage of different interferons by the therapeutics, the upstream drivers of type I IFN production, which depend not only on the cells that produce them but also the ligands that drive type I IFN. It should also be noted that even if pathogenic nucleic acid species induce much of their downstream effect through type I IFNs, there are also some IFN-independent consequences (e.g., through NF-κB activation) that may contribute to SLE, but not be blocked by IFN antibodies.

At present, it is not known whether SLE patients with an elevated type I IFN gene signature always have the same nucleic acid sensor(s) activated or if each individual patient has a unique pattern that may include any or all of them. It is also unknown if the same pathogenic nucleic acid species are present in all patients, or if they too are different from patient to patient. Furthermore, we cannot entirely exclude the possibility that the nucleic acid sensors are sufficiently dysfunctional (for any number of reasons) to trigger type I IFN production even in the absence of any aberrant DNA or RNA. However, based on the heterogeneity of SLE and the heterogeneity in response to therapeutic IFN blocking antibodies, we find it most likely that there is also heterogeneity in the presence of pathogenic DNA and RNA species resulting in the activation of a different set of sensors in each patient.

Based on GWAS and other genetic data, it also seems that a great deal of patient heterogeneity is conferred by the presence of disease-predisposing or -protective alleles in many genes, most of which are immune-related. While pathogenic nucleic acids may be instrumental in initiating and perpetuating SLE, the overall sensitivity of the immune system, as determined by all these gene variants in immune-related genes (e.g., *MHC* and *PTPN22*), will affect how readily such nucleic acids tip the balance between transient responses vs. frank autoimmune disease. Interestingly, the SLE-predisposing variant of PTPN22 not only affects T and B cell signaling, but also type I IFN production (162).

## Can SLE be Divided Into Clinically Meaningful Subpopulations Based on “Endotype”?

The unpredictable response of patients with SLE to standard of care medication is a significant challenge in rheumatology. The current paradigm is to treat patients with escalating doses of increasingly potent immunosuppressive drugs until the clinical response is deemed sufficient and then taper off the strongest immunosuppressants, particularly steroids. Even so, many patients never achieve complete remission but continue to suffer various degrees of symptoms that compromise their health and quality of life, not to mention the threat of sudden exacerbations.

In contrast, the treatment of rheumatoid arthritis made an important advance with the introduction of etanercept in 1999 (163–165). However, this drug also gave rise to the concept of “TNF-non-responders.” While most patients at least initially respond clinically to etanercept and other TNF blockers, 20–30% respond poorly, if at all. Other biologics have typically met with similar outcomes: good efficacy in many patients, but always a number of non-responders or initial responders who lose efficacy over time. It seems that non-responders represent individuals whose disease differs molecularly from the responders', such that the disease process does not involve, or readily circumvents, the specific target for the drug and therefore continues unabated.

**Figure 2:**
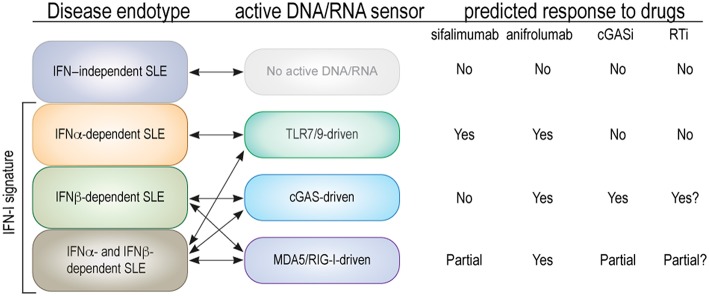
Proposed endotypes of SLE based on type I IFN subtype and relevant nucleic acid sensors. The first column represents the four proposed endotypes of SLE with double arrows connecting them to the relevant nucleic acid sensors. The predicted (or known) effects on each SLE endotype of an antibody that neutralizes IFNα only (sifalimumab), an antibody that blocks all type I IFNs (anifrolumab), and hypothetical cGAS or RT inhibitors are indicated as “yes” for a substantial clinical benefit, “no” for none, and “partial” if only one of two parallel mechanisms are expected to be inhibited.

We believe that this responder/non-responder dichotomy is also relevant in SLE and related diseases, where new drugs in recent clinical trials have generally yielded poor efficacy or a minority of (partial) responders and a majority of non-responders. As SLE is clinically highly variable, it is easy to believe that it is molecularly heterogenous as well. We propose here that SLE patients could be grouped into molecularly distinct categories (“endotypes”) based on which nucleic acid sensors are active and the IFN species produced in response to them (Figure 2):

1. IFN-independent SLE, represented by the 10–30% of patients who do not have a type I IFN gene signature and, therefore, unlikely any nucleic acid sensor activation.
2. SLE patients whose elevated and disease-driving type I IFNs are restricted to isoforms of IFNα, which are predominantly made by immune cells via TLR7/9 in response to circulating immune complexes that contain nucleic acids.
3. SLE patients with predominantly IFNβ, which is typically made by epithelial and other cells via activation of cGAS, RIG-I, or MDA5. These patients may be at an early stage of SLE development (including preclinical disease), and have not yet developed circulating immune complexes with nucleic acids. Alternatively, their disease will never develop such immune complexes.
4. SLE patients who have numerous IFNαs and IFNβ (and perhaps IFNε, IFNκ, or IFNω) and both TLR7/9 and intracellular nucleic acid sensor pathways active. If the third category includes early disease, this fourth endotype may contain severe and late stage disease.

What we propose is the personalized medicine notion that patients suffering from a disease like SLE can be subdivided by patient endotype into subsets that share a specific molecular mechanism that originally initiated and continues to perpetuate their disease, and that the inhibition of this mechanism by a selective therapeutic approach will provide a strong clinical benefit specifically to patients within this subset, but perhaps not to others. Practical examples of this concept exist in medical practice today in oncology and respiratory medicine (166) but are still absent in rheumatology. Key to the utility of this concept is the development of practical tests (“biomarkers”) that can determine which endotype individual patients belong to. In this particular case, such biomarkers would naturally quantitate nucleic acids, the activation of the sensors, and/or assess the spectrum of IFNs in patient blood or tissue.

To fully test our SLE endotype concept, future trials with new and more targeted therapeutics for SLE should include the relevant biomarkers to ask if therapeutic efficacy falls within one or another SLE endotype. In other examples of the endotype concept (166), this type of patient stratification approach has resulted in astonishing levels of efficacy within the relevant endotype, but marginal impact on patients of other endotypes. Oftentimes, these same clinical trials failed to meet their primary endpoint when the all-comers population was assessed.

## Author Contributions

TM, CL, and NG contributed equally to the writing of this review and share accountability for its content.

### Conflict of Interest Statement

The authors declare that the research was conducted in the absence of any commercial or financial relationships that could be construed as a potential conflict of interest.
